# Recent Advances in Organometallic NIR Iridium(III) Complexes for Detection and Therapy

**DOI:** 10.3390/molecules29010256

**Published:** 2024-01-03

**Authors:** Shaozhen Jing, Xiaolei Wu, Dou Niu, Jing Wang, Chung-Hang Leung, Wanhe Wang

**Affiliations:** 1 Xi’an Key Laboratory of Stem Cell and Regenerative Medicine, Institute of Medical Research, Northwestern Polytechnical University, 127 West Youyi Road, Xi’an 710072, China; jingshaoz@mail.nwpu.edu.cn (S.J.); wuxiaolei@mail.nwpu.edu.cn (X.W.); jwang0321@nwpu.edu.cn (J.W.); 2 Research & Development Institute of Northwestern Polytechnical University in Shenzhen, 45 South Gaoxin Road, Shenzhen 518057, China; 3 State Key Laboratory of Quality Research in Chinese Medicine, Institute of Chinese Medical Sciences, University of Macau, Macau 999078, China; yc17524@connect.um.edu.mo; 4 Department of Biomedical Sciences, Faculty of Health Sciences, University of Macau, Taipa, Macau 999078, China; 5 Macao Centre for Research and Development in Chinese Medicine, University of Macau, Taipa, Macau 999078, China; 6 MoE Frontiers Science Centre for Precision Oncology, University of Macau, Taipa, Macau 999078, China

**Keywords:** detection, iridium(III) complexes, NIR, therapy

## Abstract

Iridium(III) complexes are emerging as a promising tool in the area of detection and therapy due to their prominent photophysical properties, including higher photostability, tunable phosphorescence emission, long-lasting phosphorescence, and high quantum yields. In recent years, much effort has been devoted to develop novel near-infrared (NIR) iridium(III) complexes to improve signal-to-noise ratio and enhance tissue penetration. In this review, we summarize different classes of organometallic NIR iridium(III) complexes for detection and therapy, including cyclometalated ligand-enabled NIR iridium(III) complexes and NIR-dye-conjugated iridium(III) complexes. Moreover, the prospects and challenges for organometallic NIR iridium(III) complexes for targeted detection and therapy are discussed.

## 1. Introduction

Optical strategies for detection and therapy have received significant attention recently due to the advantages of minimal noninvasiveness, simple controllability, and high spatiotemporal selectivity [1]. However, traditional fluorescent probes emit in the UV–visible region, which limits their application in biomedical fields. In recent years, researchers have been researching the development of near-infrared (NIR) fluorescent probes that have emission wavelengths of over 650 nm [2,3], including cyanines, squaraines, and *azo*-boron dipyrromethenes (BODIPYs) [4,5,6]. Biological tissues exhibit low absorption and autofluorescence in the NIR region, thus achieving higher signal-to-noise ratios (SNR) [6,7]. However, organic NIR dyes suffer from limitations including aggregation, photobleaching, unspecific binding to cell components, small Stokes shifts, and poor hydrophilicity [5,8,9,10]. For example, squaraines are susceptible to be attacked by strong nucleophiles and are prone to aggregation, resulting in the formation of non-luminescent species [11]. BODIPYs have improved photostability and chemical stability under physiological conditions, but their low water solubility and their small Stokes shifts still present challenges for their use [5,9]. Indocyanine Green (ICG), the only Food and Drug Administration (FDA)-approved cyanine probe for in vivo use in medical applications, also has a small Stokes shift (λ_exc_ = 780 nm, λ_emi_ = 822 nm) and poor photostability [12,13]. Moreover, most organic fluorophores generally undergo photobleaching during long-term use, resulting in poor light stability [10,13].

Organometallic iridium(III) complexes have attracted much attention due to their superior photophysical properties, including higher photostability, tunable phosphorescence emission, long-lasting phosphorescence, high quantum yields, wide Stokes shifts, and higher photostability [14,15,16,17,18,19]. Compared with organic probes, iridium(III) complexes can avoid interference from short-lived background autofluorescence by time-resolved emission spectroscopy [20,21,22]. In addition, the modular construction of organometallic iridium(III) complexes allows for tunable properties and easy modifications [14,19,23]. A typical organometallic iridium(III) complex consists of two cyclometalated ligands and one ancillary ligand. The cyclometalated ligands regulate the wavelength of emission, allowing extension of the emission wavelength into the NIR region [14,19,23], while the modification of the ancillary ligand enables functionalization with targeting groups for precision imaging or therapy. For example, iridium(III) complexes have been developed that can visualize a variety of organelles, such as mitochondria, lysosomes, endoplasmic reticulum, Golgi apparatus, nucleus, and nucleolus, while other complexes have been demonstrated to detect specific analytes, such as small molecules, metal ions, enzymes, and proteins [16,24,25]. The unique photophysical properties of iridium(III) complexes have also been exploited for photodynamic therapy (PDT), photothermal therapy (PTT), and photoactivated (PACT) and sonodynamic therapies (SDT), largely based on non-emissive pathways of excited triplet state relaxation [26,27,28,29,30]. These characteristics of iridium(III) complexes provide myriad possibilities for the precise detection and treatment of disease. 

The ideal bioimaging probe needs to fulfill several requirements: (1) chemical stability and biocompatibility, and (2) an emission wavelength located in the NIR window in order to improve tissue penetration and improve detection sensitivity [1,5,6,18,31]. However, most phosphorescent iridium(III) complexes reported in the literature exhibit emission wavelengths below 650 nm, which hinders their clinical potential [28]. Although a number of two-photon absorption or even three-photon absorption iridium(III) complexes have been reported in the literature [32,33,34,35,36], they generally require harsh excitation conditions, expensive equipment, and narrow excitation range. Therefore, researchers have turned their attention towards the development of NIR iridium(III) complexes that have the potential to overcome the autofluorescence of biomolecules, improve tissue penetration, and reduce phototoxicity in healthy tissues [25,28,31,37].

**Figure 1:**
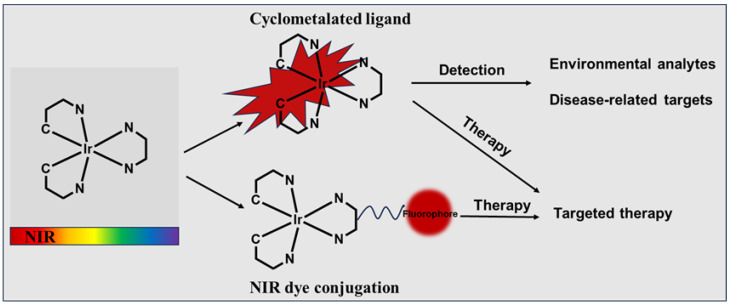
Schematic diagram of application of organometallic NIR iridium(III) complexes.

There have been several reviews on the NIR transition metal complexes [14,17,19,23,38]; however, none of them focused on the application of NIR iridium(III)-based probes for biomedical use. Therefore, this review presents the progress and strategies of organometallic NIR iridium(III) complexes for detection and therapy (Figure 1). We believe that this review will help researchers better understand the unique potential of these probes for practical applications.

## 2. General Strategies to Design NIR Iridium(III) Complexes

**Figure 2:**
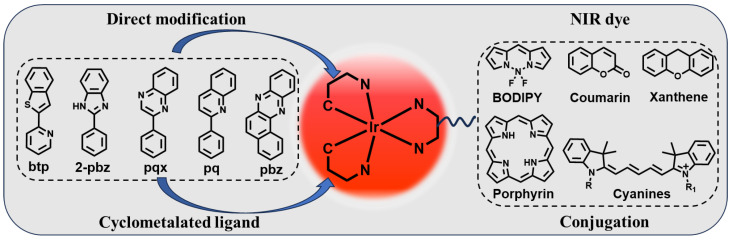
Two main strategies of designing NIR iridium(III) complexes. (1) Cyclometalated ligand-enabled NIR iridium(III) complexes. (2) NIR dye conjugation-enabled NIR iridium(III) complexes.

A typical organometallic iridium(III) complex consists of two cyclometalated C^N ligands and an ancillary N^N ligand [14,39,40]. Hence, these complexes of general structure [Ir(C^N)_2_(N^N)]^+^ are heteroleptic and monocationic, requiring a positive counterion (e.g., Cl^−^, PF_6_^−^) to balance the charge [41,42]. 2-Phenylpyridine (ppy) is the prototypical and most widely used cyclometalating ligand. The C^N ligand largely determines the strength and structural rigidity of the spin–orbit coupling (SOC) interaction; thus, it plays a dominant role in controlling the photophysical properties of the final complex [23]. Consequently, the design of the C^N ligands is critical to tailor the emission of iridium(III) complexes. To enable NIR emission, a common strategy is to elongate the π-conjugation system of the cyclometalated ligands of iridium(III) complexes by introducing electron-rich aromatic or heteroaromatic rings (Figure 2) [14,23]. Alternatively, an NIR-absorbing fluorophore moiety such as *azo*-BODIPY and cyanine can be conjugated to the iridium(III) scaffold [19,38].

A comparison of the advantages and disadvantages of these two methods is presented in Table 1.

### 2.1. Cyclometalated Ligand-Enabled NIR Iridium(III) Complexes

At present, most NIR iridium(III) complexes are constructed through the modification of cyclometalated ligands, such as 2-pyridylbenzothiophene (btp) [43,44], 2-phenylbenzimidazole (2-pbz) [40,45,46], and 2-phenylquinoxaline (pqx) [47,48]. Generally speaking, the bathochromic shift in emission wavelengths of iridium(III) complexes can be achieved by increasing the conjugation of the chelating ligands and by introducing substituents [47]. These types of NIR iridium(III) complexes have been widely developed for environmental analysis and disease-related target detection.

#### 2.1.1. Cyclometalated Ligand-Enabled NIR Iridium(III) Complexes for the Detection of Environmental Analytes

Conventional instrumental methods for detecting ions and environmental contaminants, including absorption spectroscopy and inductively coupled plasma mass spectrometry, often involve tedious preparation protocols, expensive instrumentation, long operation times, and low efficiency [49]. In recent years, luminescent transition metal complexes have been widely explored for environmental analysis and bioimaging due to their long emission lifetimes, tunable luminescence wavelengths, high photostability, and wide Stokes shifts. Consequently, considerable efforts have been undertaken to develop NIR iridium(III) complexes for the detection of environmental contaminants [50].

**Figure 3:**
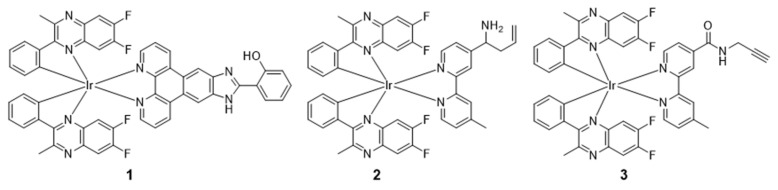
Chemical structures of **1**–**3**.

In 2020, Wang’s group reported an NIR iridium(III) complex **1** (Figure 3) to monitor boron trifluoride (BF_3_) [51,52,53]. This probe utilized 2-aryl substituted quinoxaline C^N ligands and a 2-(1*H*-imidazo [4,5-*f*][1,10]phenanthrolin-2-yl)phenol N^N ligand. The luminescence intensity of the complex at 650 nm increased in the presence of BF_3_. It is worth noting that the probe can achieve rapid detection of BF_3_ within 5 s, being faster than previously reported optical probes. Moreover, the probe allowed for the visual detection of BF_3_ on a glass pane under UV irradiation. The mechanism of detection of BF_3_ was attributed to the coordination of BF_3_ to the N and O atoms of the N^N ligand of the complex. Moreover, the probe was selective for BF_3_ over a range of common boron reagents or by-products, such as sodium tetrafluoroborate (NaBF_4_), hydrogen fluoride (HF), and boric acid (H_3_BO_3_).

The anthropogenic discharge of free metal ions can lead to human health and environmental problems. Palladium (Pd) species can bind to thiol-rich biomolecules such as amino acids and proteins, impairing the normal function of cells and even causing disease [54,55]. In 2022, Wang’s group developed the first NIR iridium(III) complex **2** (Figure 3) with allyl and amino groups in the 2,2′-bipyridine N^N ligands for imaging mitochondrial Pd species in living cells [53]. The complex displayed large Stokes shift, a long emission lifetime (314.8 ns), and good photostability. The luminescence intensity of the complexes decreased dramatically in response to Pd^0^ in solution, with a limit of detection (LOD) of 0.5 μM. In live cell imaging experiments, the probe could not only image mitochondria Pd^0^ in HeLa cells but also sense other subcellular Pd species. We hypothesized that the inherently cationic and lipophilic properties of the complex endowed it with mitochondrial specificity.

Au^3+^ ions in the environment may cause growth inhibition, immobilization, developmental malformation, and/or death of aquatic organisms. Moreover, excessive exposure to Au^3+^ ions can cause oxidative DNA damage, especially in liver, kidney, and brain tissues [56,57]. Wang’s group reported a one-step synthesis and real-time monitoring of iridium(III) complex-functionalized AuNPs, which was enabled by the reaction between the propargyl groups of an iridium(III) complex **3** and Au^3+^ ions [52]. The probe exhibited multimodal characteristics, which decreases interference of other metal ions, thereby increasing selectivity of the probe for Au^3+^ ions. Moreover, as the Au^3+^ concentration increased from 0.5–200 μM, the emission intensity gradually decreased at 470 nm and 700 nm. A linear relationship between luminescence quenching at 700 nm and the concentration of Au^3+^ ions was observed in the range of 1–15 μM, with a detection limit of 0.38 μM. The photophysical properties of complexes **1**–**3** are summarized in Table 2.

#### 2.1.2. Cyclometalated Ligand-Enabled NIR Iridium(III) Complexes for the Detection of Disease-Related Targets

Fluorescent probes have been widely used in biological imaging for disease diagnosis [58,59,60]. Due to their desirable advantages [61,62,63], the study of NIR iridium(III) complexes for biological imaging, sensing, and cancer diagnosis is rapidly increasing.

**Figure 4:**
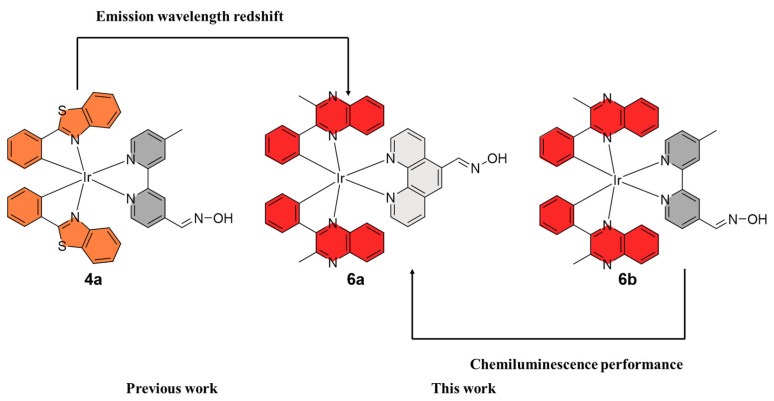
Construction of the multimodal HClO imaging probe. Reproduced with permission from Ref. [66] Copyright 2023 American Chemical Society.

**Figure 5:**
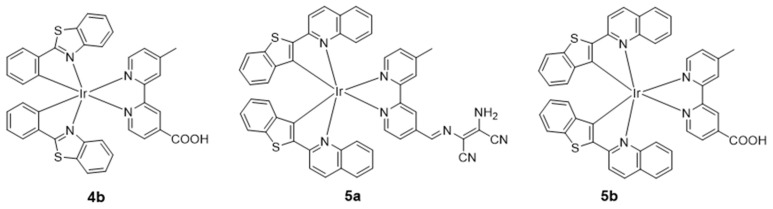
Chemical structures of **4b**–**5b**.

In 2020, Lv’s group developed an oxime-decorated iridium(III) complex **4a** as a multimodal imaging probe for simultaneous chemiluminescence (CL) as well as two-photon luminescence lifetime imaging of hypochlorous acid (HClO) in living systems (Figure 4) [64]. Later, by extending the conjugation of the aromatic rings of the previous complex, the authors developed the oxime-conjugated iridium(III) complex **5a** (Figure 5) as the first example of a two-photon NIR probe for multisignal detection and multimodal imaging with ClO^−^ [65]. After the reaction with ClO^−^, the maximum emission intensity at 663 nm was significantly increased. Most recently, this group synthesized an ionic iridium(III) complex **6a** by using the electron-rich 2-phenyl-3-methylquinoxaline as the C^N ligand and an oxime-modified N^N ligand to afford a molecular NIR CL probe. The CL signals of NIR **6a** showed a good linear relationship with HClO concentrations within the range of 1–80 μM. Probe **6a** was highly sensitive for HClO detection, while the LOD at 0.14 μM (3σ/k) was lower than most reported detection methods [66].

Biothiols mainly include three biomolecules, cysteine (Cys), homocysteine (Hcy), and glutathione (GSH). Aberrant levels of biothiols are indicative of different pathological conditions, including cardiovascular diseases, liver injuries, and cancer. Moreover, thiols affect oxidative stress in cells and are involved in the production of signal molecules (such as H_2_S), protein functionalization, and lipid metabolism [67].

**Figure 6:**
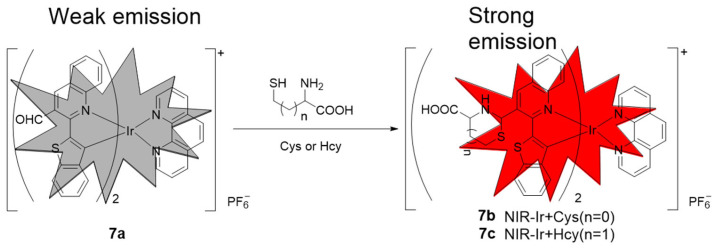
Schematic illustration of the mechanism of **7a** response to Cys and Hcy. Red simply represents enhanced luminescence of the complex. Reproduced with permission from Ref. [68]. Copyright 2017 Royal Society of Chemistry.

**Figure 7:**
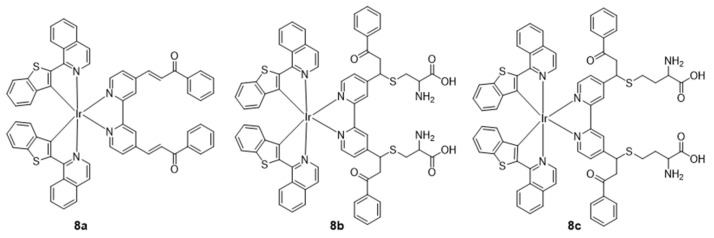
Chemical structures of **8a**–**8c**.

In 2017, Fan’s group first reported an NIR iridium(III) complex with an aldehyde group on the main C^N ligand btp for visualizing Cys and Hcy in live cells and mice [68]. In the presence of Hcy/Cys, the luminescence intensity of **7a** is enhanced, which is due to the fact that the electron-withdrawing property of the aldehyde group is abolished by reaction with the thiol (Figure 6). The detection limit was 13.7 μM for Cys and 9.7 μM for Hcy (S/N = 3), respectively. In 2020, the same group reported another NIR iridium(III) complex **8a** with the main C^N ligand btp for visualizing Cys and Hcy in live cells and mice, in which the α,β-unsaturated ketone group, which acts as a quencher, is connected to the ancillary ligand ppy (Figure 7) [69]. The nucleophilic addition reaction between **8a** and aminothiol occurred rapidly and the emission intensity increased by more than 40 times. In 2022, Lo’s group reported NIR iridium(III) polypyridine methylsulfone complexes **9** with high reactivity and selectivity towards Cys-bearing peptides and proteins [70]. The emission of the complex was regulated by using the highly π-conjugated ligand benzo[*a*]phenazine (bpz).

Peroxynitrite (ONOO^−^), a highly reactive nitrogen species (RNS) in the biosphere, is produced by the diffusion-controlled reaction between nitric oxide (NO) and superoxide (•O^2−^) radicals. It plays an important role in activating or inducing biological signal transduction processes and immune responses, as well as modulating redox homeostasis. An excessive amount of ONOO^−^ is associated with cancer, cardiovascular diseases, and neurodegenerative disorders [59,71].

**Figure 8:**
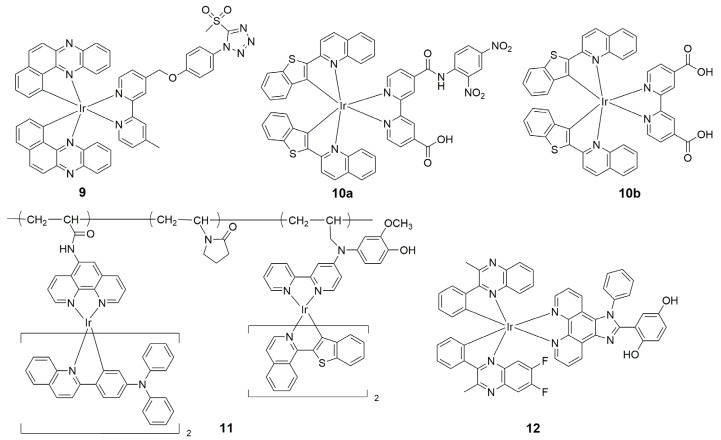
Chemical structures of **9**–**12**.

In 2019, a novel NIR iridium(III) complex was synthesized to detect ONOO^−^ within seconds (Figure 8) [72]. A strong electron-withdrawing group, 2,4-dinitroaniline, was introduced into the accessory ligand of **10a**, which reacts with the oxidizer ONOO^−^. Importantly, probe **10a** showed an emission wavelength in the NIR region between 660 nm and 710 nm and was able to specifically sense ONOO^−^ produced in living cells and mouse models of inflammation.

In 2020, Zhao’s group developed a luminescence method for exploring acute drug-induced liver injury in vivo based on a phosphorescent polymer probe **11** with NIR dual-emissive characteristics [73]. With the utilization of **11**, the produced ONOO^−^ was visualized successfully in drug-treated hepatocytes with a high signal-to-noise ratio via ratiometric and time-resolved photoluminescence imaging. Encouragingly, the increase in ONOO^−^ produced in ketoconazole-induced liver injury was directly observed for the first time [73].

In 2021, Chao’s group developed a mitochondria-targeted NIR iridium(III) complex with an approximate maximum emission wavelength at 704 nm [74]. It has redox reversible properties and was used for the detection and imaging of cellular redox status by visualizing endogenous ONOO^−^/GSH. Probe **12** was successfully applied to monitor the reversible redox cycle between ONOO^−^ and GSH in living animals, which means that **12** has great potential to be utilized for evaluating hepatotoxicity caused by drugs such as acetaminophen (APAP) and the progress of therapy by drugs such as *N*-acetylcysteine (NAC) in animals. The photophysical properties of complexes **4a**–**12** are summarized in Table 3.

**Figure 9:**
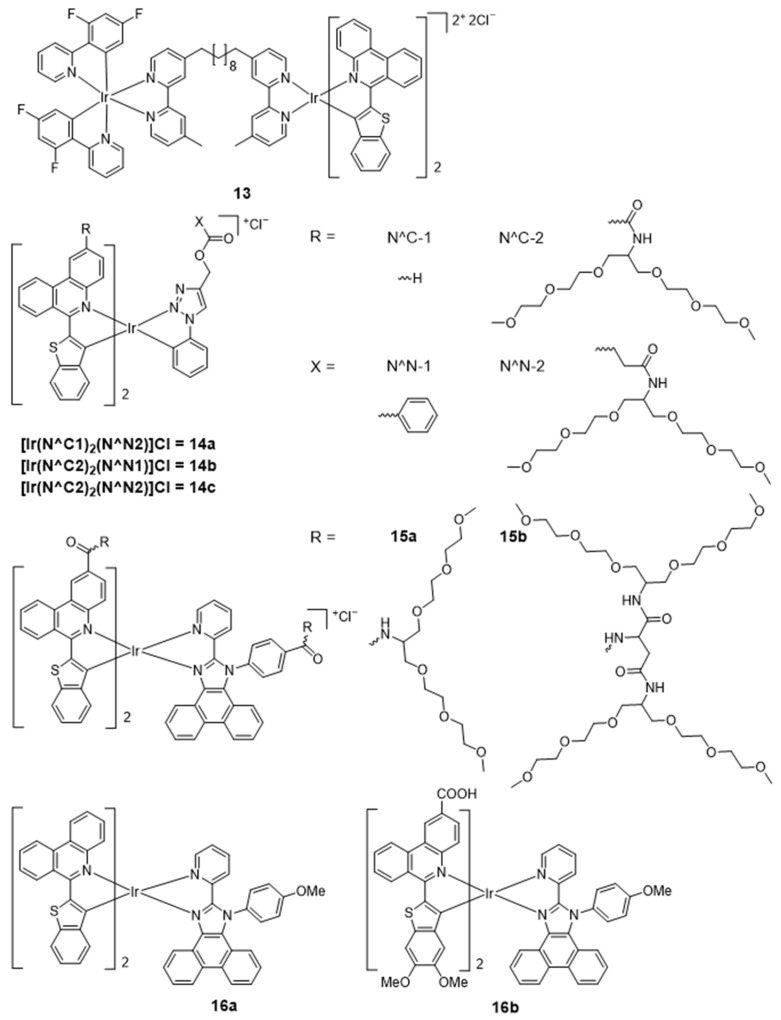
Chemical structures of **13**–**16b**.

Biological microviscosity is one of the most essential micro-environmental parameters and contributes to biological functions by affecting the interaction and transportation of biomolecules and chemical signals within live cells. In 2018, Sun’s group reported a luminescent bimetallic iridium(III) complex for ratiometric tracking intracellular viscosity [75]. Probe **13** (Figure 9) has a large Stokes shift of 258 nm and exhibits dual emission maxima at around 521 and 708 nm. More importantly, **13** is cell-permeable and can be employed to distinguish cancer cells from normal cells and track viscosity changes during MCF-7 cell apoptosis.

Oxygen is an important regulator of normal cells and an important indicator of cell/tissue physiological status under healthy and diseased conditions [76,77]. The demand and supplement of oxygen is thought to be closely linked to a variety of serious diseases, such as cancers, neurological diseases, arteriosclerosis, cerebral infarction, ischemic heart disease, chronic kidney disease, and diabetic retinopathy. The monitoring of oxygen concentration in the body can effectively diagnose and trace the metastasis of pathological tissues as well as more accurate personality therapy [78,79].

At present, the most widely used ligand in oxygen sensing is 2-(2-pyridyl)benzothiophene or related C^N ligands such as phenanthridine-benzothiophene (btph) cyclometalating ligand. In 2020, Kritchenkov et al. reported three iridium(III) complexes (**14a**–**14c**) bearing benzothienyl-phenanthridine groups as the cyclometalating C^N ligands and a pyridine-triazole ligand as the ancillary N^N ligand. With extended conjugation systems, these complexes had intense phosphorescence emission bands with maxima in the range of 710–720 nm [76]. Subsequently, a series of iridium(III) complexes (**15a**–**15b**) with the benzothienyl-phenanthridine group were further constructed [77]. All the complexes are luminescent in aqueous media with emission in the NIR region (~730 nm), a high quantum yield of up to ca. 12% in degassed solution, wide Stokes shifts, and lifetimes in the microsecond domain (3.23–3.28 μs). In the following report, a range of [Ir(N^C)_2_(N^N)]^+^-type iridium(III) complexes were synthesized, where the C^N cyclometalating and N^N diimine ligands were varied to modulate the donor/acceptor ability of substituents. These complexes displayed strong NIR emission (λ_emi_ > 710 nm) and high quantum yields from 10.3 to 20.5% in degassed methanol. All the complexes showed strong lifetime dependence on oxygen concentration, and two of them (**16a**–**16b**) were utilized as oxygen sensors in cell cultures [80]. The photophysical properties of complexes **13**–**16b** are summarized in Table 4.

#### 2.1.3. Cyclometalated Ligand-Enabled NIR Iridium(III) Complexes for Targeted Therapy

Iridium(III) complexes have attracted great interest as promising photosensitizer (PS) candidates because of their favorable chemical and photophysical properties, long excited lifetimes, and high intersystem crossing (ISC) ability. As PSs, iridium(III) complexes exhibit highly populated triplet states for ROS production that can be tuned by modifying the coordination of different types of ligands. However, the long-wavelength excitation and deeper tissue penetration of PSs are crucial to ensure effective therapeutic effects. Unfortunately, most of the reported cyclometalated iridium(III) complexes require activation by short wavelengths, which compromises their potential clinical application. To shift the absorption and emission wavelengths of iridium(III) complexes to longer wavelengths, electron-donating or electron-withdrawing substituents can be introduced at appropriate positions on the aromatic ring of the ligands [81].

**Figure 10:**
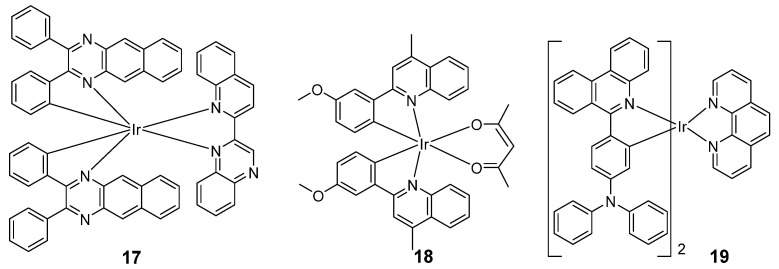
Chemical structures of **17**–**19**.

In 2017, Sun’s group synthesized five heteroleptic cationic iridium(III) complexes as in vitro theranostic PDT agents, with an π-expansive cyclometalating 2,3-diphenylbenzo[*g*]quinoxaline (dpbq) ligand as the C^N ligand [48]. The maximum emission wavelengths of these five iridium(III) complexes were 910–916 nm upon excitation at 473 nm due to the extensive degree of π-conjugation of the C^N ligands. All the complexes were biologically active toward melanoma cells in vitro. Complex **17** (Figure 10) became a very potent cytotoxin with light activation, with photocytotoxicity indexes (PIs) of almost 275 and EC_50_ values as low as 12–18 nM. These iridium(III) complexes induced aggregation of DNA and production of ^1^O_2_ in cell-free experiments, and were taken up readily by melanoma cells. However, their precise intracellular biological target(s) and mechanism(s) of action remain unknown.

In 2022, Tao et al. reported two new series of iridium(III) complexes based on the pq ligand by simply introducing a methoxyl group into different positions of the metalated phenyl moiety [82]. The maximum absorption peaks of **18** were located at 660 nm (Φ_PL_ = 0.40 and Φ_Δ_ = 0.73) in oxygen-free dimethyl sulfoxide (DMSO). The complex showed high sensitivity to oxygen, with cell images under 2.5% O_2_ being brighter than those taken under 21% O_2_. The hypoxia imaging performance of **18** was then evaluated in vitro. A strong phosphorescence (ROI_B_ = 1.707 × 10^10^) was observed from tumor tissues under hypoxia, while a relatively weak phosphorescence (ROI_A_ = 6.366 × 10^9^) was detected from the subcutaneous tissue of nude mouse. Moreover, the complex was also applied for PDT in HepG2 cells. In 2023, He’s group developed a new NIR AIE-active iridium(III) photosensitizer via attaching triphenylamine on the 6-phenylphenantridine for mitochondria-targeted cancer PDT [83]. Complex **19** emits weakly in pure acetonitrile, but, as the volume fraction of water increases, the NIR phosphorescence (ca. 650–750 nm) is gradually enhanced with typical AIE features (Figure 10). In an in vivo cancer study, the tumor volumes of mice treated with **19** were decreased by about 59.8%, showing a strong PDT antitumor effect. Moreover, mechanistic studies revealed that **19** caused cellular ROS overproduction, mitochondria dysfunction, and ER stress in MDA-MB-231 cells upon photoirradiation, leading to apoptotic cell death. Overall, **19** is a promising PS for mitochondria-targeted imaging and cancer phototherapy.

**Figure 11:**
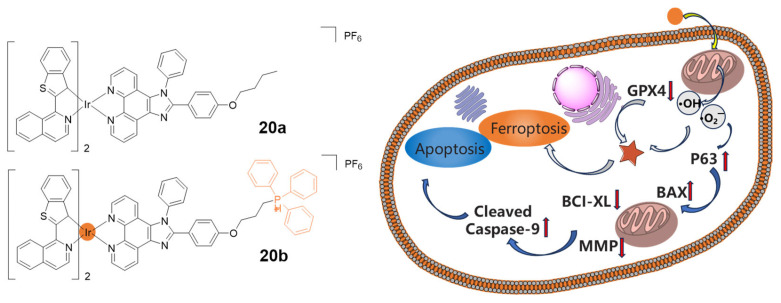
Chemical structures of complexes **20a** and **20b**, and the illustration of cell death pathways induced by 20b. Reproduced with permission from Ref. [84]. Copyright 2021 John Wiley and Sons (Hoboken, NJ, USA).

In 2021, complexes **20a** and **20b**, two new benzothiophenylisoquinoline (btiq)-derived cyclometalated iridium(III) complexes, were reported by the He group (Figure 11) [84]. Indeed, **20a** and **20b** exhibited maximum absorption wavelengths at 488 nm, while the maximum emission wavelengths were at 685 nm. Both complexes were active in type I PDT processes, generating hydroxyl radicals (•OH) and superoxide radicals (•O^2−^) in hypoxic conditions. Complex **20b** accumulated preferentially in mitochondria due to introducing the presence of the mitochondria-targeting triphenylphosphonium group. Importantly, the PI of **20b** under hypoxia was 3.6 times higher than **20a** because of its mitochondria-targeting ability. Mechanistically, **20b** combined the effects of ferroptosis and apoptosis to exert a dual mode of cell death, via both inhibiting ATP production and inducing more distinct mitochondria morphological change. Overall, the synergism of ferroptosis and apoptosis offered a new way to combat hypoxic and apoptosis-resistant tumor cells.

**Figure 12:**
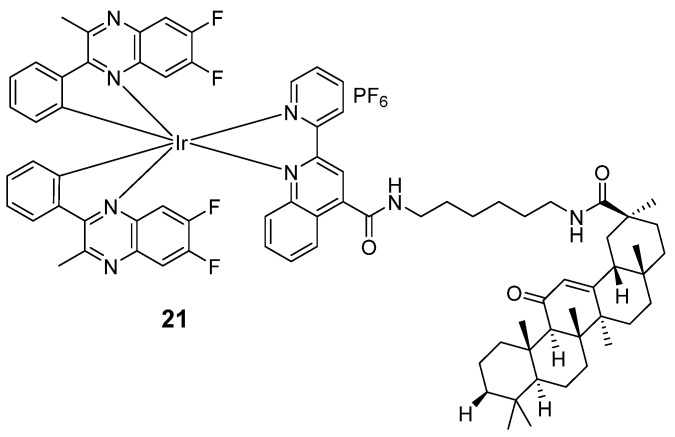
Chemical structure of **21**.

Recently, Wang et al. developed an NIR luminescent theranostic **21** (Figure 12) for hepatocellular carcinoma (HCC) diagnosis and treatment through conjugating an iridium(III) complex to glycyrrhetinic acid (GA). The maximal emission peak of **21** occurred at around 686 nm by employing pqx-type C^N ligands. With the conjugation of GA, complex **21** could be selectively taken up by HepG2 liver cancer cells, and **21** (IC_50_ = 1.26 ± 0.07 μM) exhibits superior antitumor activity of its ligand GA (IC_50_ = 39.81 ± 0.2 μM) by enhancing mitochondrial targeting. Mechanistic studies revealed that **21** could target mitochondria and induce ROS accumulation, increase mitochondrial membrane permeability, and increase the Bax/Bcl-2 ratio to promote apoptosis of HCC cells. Moreover, complex **21** could distinguish HCC cells from other cells via NIR imaging. This work paves the way for the development of multifunctional probes that integrate diagnosis and therapeutics [85]. The photophysical properties of complexes **17**–**21** are summarized in Table 5. 

### 2.2. NIR Dye Conjugation-Enabled NIR Iridium(III) Complexes

Recently, an increasing number of transition metal complexes, such as Pt(II), Ru(II), Os(II), and iridium(III) complexes, have been explored for treatment by introducing fluorescent groups, such as BODIPY [86], porphyrin [87], xanthene [88], coumarin [89], and cyanine derivatives [90]. By taking advantage of fluorescent π–π* transitions at long wavelengths and cyclometalated iridium(III) complexes with efficient ISC and a long-lived triplet excited state, their conjugation can be exploited for developing theranostic agents. It is worth noting that no obvious wavelength shift in the conjugation is observed compared to the fluorescent groups, meaning that the optical properties of the conjugates mainly depend on the complexation of organic fluorophores. This also implies that the molecular orbitals of the ligand remain largely unchanged during the complexation process [86].

#### NIR Dye Conjugation-Enabled NIR Iridium(III) Complexes for Phototherapy

Among organic fluorophores, BODIPY, porphyrin, coumarin, rhodamine, and phthalocyanine derivatives have become important tools in diagnoses and therapy because of their high molar absorption coefficients and long-wavelength excitation [91,92]. However, their poor ISC capacity leads to lower ROS production, resulting in unsatisfactory PDT treatment [93]. In this context, it is a promising strategy to combine the rich and tunable photophysical anticancer properties of transition metal complexes with the photochemical properties of organic small-molecule chromophores. As a result, long-wavelength excitation and long-lived triplet metal-to-ligand charge transfer (^3^MLCT) states can be obtained for high ROS generation.

**Figure 13:**
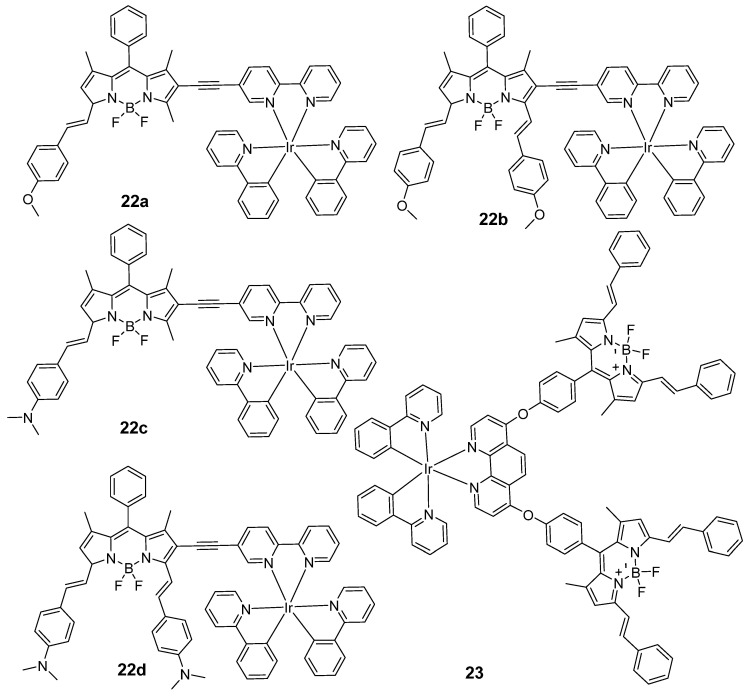
Chemical structures of **22a**–**23**.

During the past decade, BODIPY has proven to be an attractive PS to be introduced into transition metal complexes [86,94,95]. In 2014, Zhao’s group developed a method to obtain transition metal complexes with strong visible light absorption and long-lived triplet excited states by attaching BODIPY to the coordination center via a π-conjugation linker (C-C triple bond) [86]. As a result, the complexes **22a**–**d** (Figure 13) showed strong NIR absorption (644–729 nm), strong NIR fluorescence (700–800 nm), and long-lived triplet excited states (92.5–156.5 μs) under N_2_. The maximum emission peak of **22c** (683 nm) was red-shifted compared to **22a** (624 nm), which was attributed to superior intramolecular charge transfer (ICT) effects between BODIPY and the styryl group with an amino substituent. Similar trends were observed for the distyryl compounds **22b** and **22d.** Another series of BODPIY-linked iridium(III) complexes were reported in 2019 [96]. The UV–vis absorption of complex **23** was obtained in the red region of the spectrum with high molar extinction coefficients (19.49 × 10^4^ M^−1^ cm^−1^). Unfortunately, the production of triplet excited states was not supported, which could have been due to the inappropriate distance between the coordination center and BODIPY unit. Nonetheless, the value of φ_Δ_ for **23** was six times higher than that of the free BODIPY.

**Figure 14:**
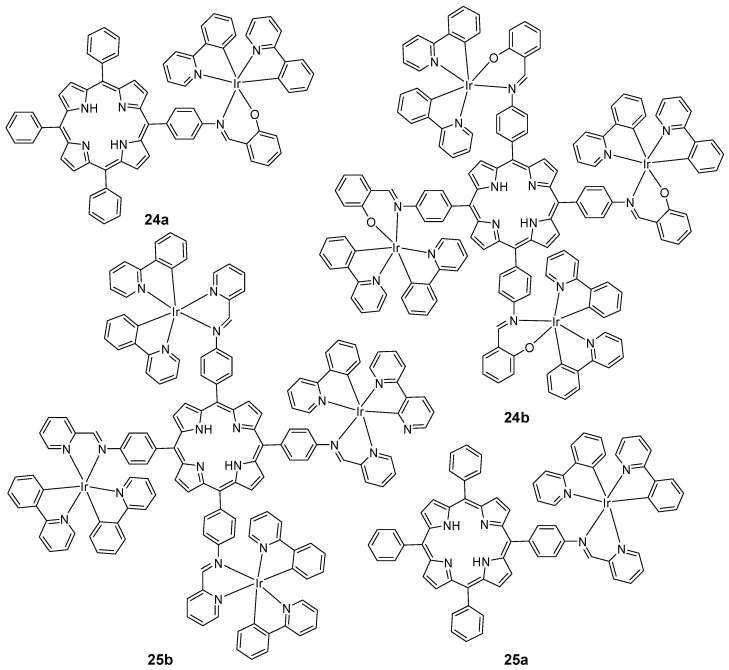
Chemical structures of **24a**–**25b**.

Due to their absorption in the red-light region, porphyrin derivatives have been utilized as phototherapy agents. In 2021, Bryce’s group combined the respective advantages of small organic molecules and transition metal complex PSs to obtain iridium(III)-porphyrin conjugates **24a**–**24b** (Figure 14) with long-wavelength excitation for high-efficiency synergistic PDT and PTT treatment [87]. The complexes possessed deep-red absorbance, long-wavelength excitation (635 nm), and NIR emission (720 nm). With the increasing number of iridium(III) centers from tetraphenylporphyrin (TPP), the HOMO–LUMO energy gap decreases. The relatively narrow gap suggests a long-wavelength absorption, especially for **24b**, which is promising for phototherapy. Meanwhile, **24b** possessed higher photothermal conversion efficiencies (PCE) compared with **24a** (49.5% vs. 37.8%), indicating that the additional iridium(III) centers can improve the thermal effect. Subsequently, this group reported mono- and tetra-nuclear iridium(III) complex-porphyrin conjugates that exhibited long wavelength absorption (500–700 nm) and NIR emission (635–750 nm) [97]. Similar to the previous results, the introduction of additional iridium(III) centers to extend the π-conjugation could enhance the PDT effect. In measurements of time-dependent kinetics of ^1^O_2_ generation, the kinetic decay of **25b** was 55.5 times that of **25a**. Finally, complex **25b** exhibited obvious AIE characteristics and low half-maximal inhibitory concentration against HeLa cells (IC_50_ = 0.47 × 10^−6^ M).

**Figure 15:**
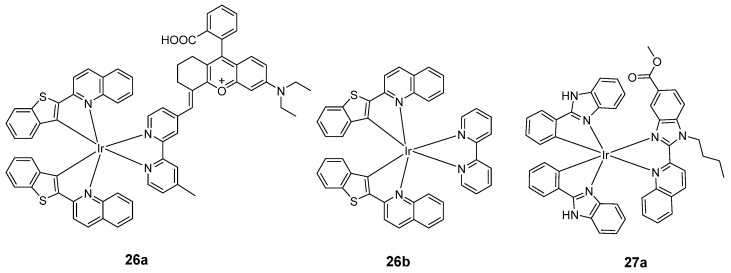
Chemical structures of **26a**–**27a**.

Xanthene dye, one of the most common organic dyes, has been widely applied in chemosensors and biomolecules because of its excellent photophysical properties and high mitochondria-targeting ability [98]. In 2021, Wong’s group reported a mitochondria-targeting PS iridium(III) complex **26a** (Figure 15) by introducing xanthene dye [88]. The complex exhibits synergistic PDT effects, including low dark cytotoxicity, selective mitochondria-targeting uptake, high molar absorptivity, and high photostability. The emission spectrum of **26a** showed an intense emission peak at around 650 nm, with a shoulder at around 705 nm. The generated quantum yield for ^1^O_2_ of **26a** was 0.72, which is much higher than that of **26b** (0.29) and its N^N ligand, thus improving the problem of low ^1^O_2_ generation efficiency. This result indicates that the combination of the xanthene dye and phosphorescent iridium(III) center exhibits synergistic merits for PDT applications. Moreover, **26a** shows stronger mitochondria-targeting properties due to the introduction of xanthene dye. Mechanistically, **26a** induced mitochondrial depolarization and apoptosis. The in vivo photo-antitumor activity of the complex was further demonstrated in tumor-bearing mice.

Another challenging problem that a potential PS faces is the treatment of solid tumors under highly hypoxic conditions [99,100,101]. Therefore, there is an urgent need to overcome the shortcomings of hypoxia and achieve more ideal tumor treatment. From the perspective of the therapeutic mechanism of PDT, type II PDT produces singlet oxygen (^1^O_2_) by direct energy transfer from PS to the ground state of molecular oxygen. On the other hand, type I reactions generate several other cytotoxic reactive species, such as OH• and •O^2−^, following photoinduced electron transfer [102,103]. Therefore, type I PDT overcomes the intrinsic limitations of conventional PDT treatment owing to the diminished O_2_ dependence and achieves superior solid tumor PDT efficacy [104]. An alternative strategy to generate singlet oxygen is the use of sonosensitizers. The benefit of sonosensitizers is that ultrasound exhibits much greater depth in tissue penetration compared with light. Moreover, sonosensitizers can be combined with photoacoustic imaging to guide ultrasound irradiation time during treatment. Recently, iridium(III)-phthalocyanine and iridium(III)-cyanine complexes have been reported for sonosensitizer and photoacoustic imaging applications [90,105].

**Figure 16:**
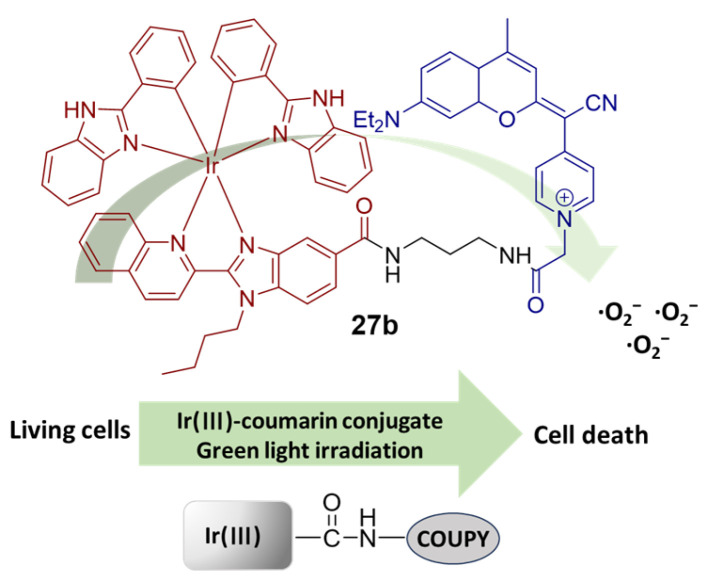
Schematic representation of the iridium(III)-coumarin conjugate **27b**. Reproduced with permission from Ref. [89]. Copyright 2019 John Wiley and Sons.

In 2019, Marchán’s group reported a cyclometalated iridium(III) complex **27b** (Figure 16) conjugated to a far-red-emitting coumarin for cancer phototherapy [89]. The ^1^O_2_ quantum yield of coumarin (<0.01) increased by one order of magnitude in the complex **27b** (>0.19) in all organic solvents due to an enhanced ISC induced by the heavy iridium(III) ion. Treatment with **27b** generates a specific type I ROS in living cells upon visible-light irradiation, •O^2−^, overcoming the drawback of traditional PSs such as O_2_-tension dependency. The low dark cytotoxicity of **27b** led to excellent PIs of 85 and 161 after irradiation with green and blue light, respectively. Moreover, the cytotoxicity of compounds **27a** and **27b** was similar in both normoxic and hypoxic conditions.

**Figure 17:**
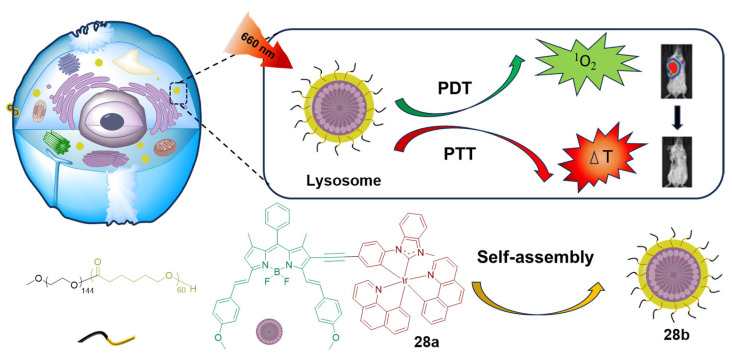
Schematic illustration of the working mechanism of iridium(III) complex-derived polymeric micelles for combined PDT and PTT. Reproduced with permission from Ref. [32]. Copyright 2021 John Wiley and Sons.

According to the energy gap law [106], the low-energy excited state associated with low energy absorption will sharply increase the non-radiative decay rate of thermal relaxation. However, enhanced non-radiative relaxation may produce hyperthermia for PTT, which could be exploited to kill hypoxic tumors. In 2021, Chen’s group developed an NIR iridium(III) complex (**28a**) for potent PDT/PTT (Figure 17) [32]. By the combination of the neutral iridium(III) complex with the BODIPY scaffold, the population of the triplet excited state in **28a** is increased, with enhanced non-radiative decay. The iridium(III) complexes absorb strongly at 550–750 nm with a band maximum at 685 nm. Upon micellization, **28a** forms *J*-type aggregates (**28b**). Due to the high molar extinction coefficient and the amplification of light-to-ROS/heat conversion, the generation of ^1^O_2_ and photothermal effects are promoted, causing severe apoptosis. Aggregate **28b** not only destroyed orthotopic 4T1-Luc tumors but also prevented metastasis to the lung damage under light irradiation, manifesting potent photocytotoxicity via synergetic PDT/PTT damage.

**Figure 18:**
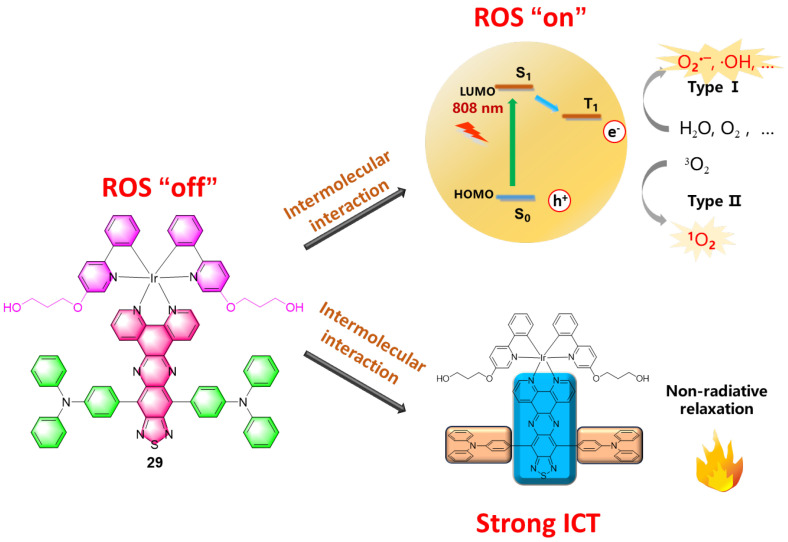
The structure and anticancer mechanism of **29** for NIR I-type PDT and PTT are depicted schematically. Reproduced with permission from Ref. [108]. Copyright 2020 John Wiley and Sons.

In 2020, Gou’s group combined an iridium(III) complex with a donor–acceptor–donor (D–A–D)-type ligand to fabricate complex **29** (Figure 18) for NIR I-type PDT and PTT [107]. By using triphenylamine (TPA) and [1,2,5]thiadiazolo-[3,4-*i*] dipyrido[*a,c*]phenazine (TDP) as the electron donor and acceptor, **29** showed evident NIR absorption in the 600–1000 nm region, with an absorption maximum at 716 nm (ε = 9.0 × 10^3^ M^−1^ cm^−1^), which was assigned to an ICT transition. The maximum absorption peak of **29** was gradually red-shifted to 814 nm with an increase in the water fraction up to 95%. Moreover, the significant ICT of the D–A–D chromophores also endowed a nonradiative deactivation pathway from the singlet excited state for heat generation. The robust heat generation capabilities are reflected in the high PCE of 27.5% and 34.9% for **29** and **29**-**NPs,** respectively, making them superior to photothermal gold nanorods (e.g., ≈21.0%). Complex **29** was also conjugated with PEG and formulated into nanoparticles (**29**-**NPs**), which preferentially accumulated in the tumor area and showed a significant in vivo tumor regression (96%) through synergistic PDT and PTT. The photophysical properties of complexes **22a**–**29** are summarized in Table 6.

## 3. Conclusions and Perspective

Iridium(III) complexes have found widespread applications in targeted detection and therapy. However, their limited ability to be excited by longer-wavelength light and emit in the longer wavelength region has hindered their development. To overcome this limitation, there are two general strategies for the development of NIR probes. One important strategy to enable NIR properties in iridium(III) complexes is to extend the π-conjugation of the cyclometalated ligands or introduce electron-rich heteroaromatic rings. Nevertheless, the availability of cyclometalated ligands for NIR iridium(III) complexes is limited as most complexes are constructed using a few cyclometalated ligands, such as btp, pbz, and 2,3-disubstituted quinoxaline. Therefore, exploring new cyclometalated ligands is urgently required. Additionally, some iridium(III) complexes may encounter solubility issues due to the introduction of large π-conjugated cyclometalated ligands, such as pqx. Furthermore, although iridium(III) complexes can emit in the NIR region, the challenge of requiring excitation by short-wavelength light remains unsolved, limiting their application in therapy due to potential damage and poor tissue penetration of short-wavelength light. The second strategy to enable NIR properties in iridium(III) complexes involves attaching NIR-absorbing fluorophore units such as BODIPY, cyanine, porphyrin, and coumarin to iridium(III) complexes. This approach enables a change in the excitation and emission wavelengths, potentially increasing the quantum yield. Moreover, the introduction of NIR-absorbing fluorophore units may offer a novel mechanism of action. However, it should be noted that this strategy often requires complicated synthesis. As discussed in this review, organometallic NIR iridium(III) complexes have made significant progress as versatile probes in environmental and biological detection, including the detection of environmental analytes and disease-related targets. Some NIR iridium(III) complexes have even demonstrated success in vivo on tumor-bearing mice, particularly those conjugated with NIR dyes.

Studies on the therapeutic applications of NIR iridium(III) complexes still remain relatively rare. Some NIR iridium(III) complexes have been applied for therapy, which mainly rely on type II photochemical processes to generate singlet oxygen (^1^O_2_). Unfortunately, this pathway heavily depends on the oxygen concentration in tumors, leading to undesirable efficacy. Alternatively, type I photochemical processes involving electron transfer mechanisms present a viable option for NIR iridium(III) complexes. Azo-BODIPY, for instance, has shown promise in photogenerating •OH and •O^2−^. Additionally, NIR dyes possess low-lying excited states associated with low-energy absorption, which can increase non-radiative decay rates, leading to hyperthermia for PTT. Combining PDT and PTT using NIR iridium(III) complexes could introduce new strategies for cancer treatment.

In summary, the development of NIR iridium(III) complexes for detection and therapy deserves further exploration. It is critical to develop iridium(III) complexes with longer wavelength excitation and emission for in vivo applications. In this context, fluorophores emitting in the second near-infrared region (NIR-II, 1000–1700 nm) have gained recent attention for biosensing, bioimaging, and phototherapy. Thus, the fabrication of NIR-II iridium(III) complexes could substantially expand their in vivo applications. Another challenge faced by NIR iridium(III) complexes is poor tumor targetability. Incorporating tumor-targeting units into metal complexes is a viable strategy to improve the targetability of metallodrugs [108,109], which could provide the basis for developing targetable NIR iridium(III) complexes. However, NIR iridium(III) complexes generally have more complicated structures, and the introduction of targeting moieties could increase molecular weight, which may decrease aqueous solubility and impair their bioavailability. Thus, more efforts need to be undertaken for designing targetable NIR iridium(III) complexes with simpler structures and lower molecular weight. Furthermore, the self-assembly approach is an emerging strategy to construct multifunctional molecules in situ [110]. This could be adapted for developing novel tumor-targeting NIR iridium(III) complexes with desirable photophysical and physicochemical properties for precise detection and therapy. Considering the rapid technological advancements in organometallic NIR iridium(III) complexes, we anticipate that they have the potential to become mainstream tools for targeted detection and therapy in the future. This will facilitate their rapid development in environmental analytes, disease-related targets, therapy, and other biomedical fields.

## Figures and Tables

**Table 1:** The advantages and disadvantages of two methods (direct modification with cyclometalated ligand and indirect modification with NIR dye).

	Direct Modification with Cyclometalated Ligand	Indirect Modification with NIR Dye
Advantages	⬤ Easy synthesis ⬤ Large Stokes shifts ⬤ Tunable emission wavelength ⬤ Ease of N^N ligand modifications for functionalization ⬤ Suitable for detection	⬤ High ROS generation ⬤ Low-energy excitation wavelength ⬤ Long emission wavelength for better tissue penetration ⬤ Suitable for therapy
Disadvantages	⬤ Short excitation wavelength ⬤ Lack of cyclometalated ligands ⬤ Poor water solubility	⬤ Complicated synthesis ⬤ Small Stokes shifts ⬤ Difficult to tune excitation and emission wavelength

**Table 2:** Photophysical properties of **1**–**3**.

Complexes	Target	Solvent	λ_abs_/nm	λ_emi_/nm	Φ_PL_	Lifetime/ns	Ref.
**1**	BF_3_	ACN	280 (DMF)	475, 650	0.24	356.0	[51]
**2**	Pd species	ACN	260	670	0.0641	314.8	[53]
**3**	Au^3+^	ACN	365	700	0.0606	368.8	[52]

**Table 3:** Photophysical properties of **4a**–**12**.

Complexes	Target	Solvent	λ_abs_/nm	λ_emi_/nm	Φ_PL_	Lifetime/ns	Ref.
**4a**	HClO	DMF/PBS	318, 408	/	0.00002	3.78	[64]
**4b**	/	DMF/PBS	310, 408	540, 570	0.00178	369.1	[64]
**5a**	ClO^−^	MeOH/PBS	284, 398, 502	663	0.002	165	[65]
**5b**	/	MeOH/PBS	285, 361,498	662	0.104	382	[65]
**6a**	HClO	DMF/PBS	370, 472	663	0.00076	/	[66]
**6b**	/	DMF/PBS	369, 470	663	0.00052	/	[66]
**7a**	Cys/Hcy	EtOH	261, 345,500	680	0.008	158	[68]
**7b**	/	EtOH	261, 345, 500	670	0.018	/	[68]
**7c**	/	EtOH	261, 345, 500	670	0.021	/	[68]
**8a**	Cys/Hcy	DMSO/PBS	486, 303	683, 748	0.005	/	[69]
**8b**	/	DMSO/PBS	286, 310, 358, 486	683, 748	0.109	/	[69]
**8c**	/	DMSO/PBS	285, 311, 358, 486	683, 748	0.122	/	[69]
**9**	Cys-bearing peptides and proteins	ACN	/	668	0.048	346	[70]
**10a**	ONOO^−^	DMSO/PBS	289, 320, 360, 500	660, 710	0.012	7490	[72]
**10b**	/	DMSO/PBS	289, 324, 350, 500	660, 710	0.131	7140	[72]
**11**	ONOO^−^	H_2_O	/	605, 678/743	0.04	600/680	[73]
**12**	ONOO^−^/GSH	DMSO/PBS	302	704	0.136	/	[74]

**Table 4:** Photophysical properties of **13**–**16b**.

Complexes	Solvent	λ_abs_/nm	λ_emi_/nm	Φ_PL_	Lifetime/ns	Ref.
**13**	ACN	/	521, 708	0.063 (H_2_O)	1770	[75]
**14a**	MeOH	331, 373, 393, 424, 496, 524	710, 775, 881, 943	0.014	270	[76]
**14b**	MeOH	336, 374, 390, 433, 511, 534	719, 782, 882, 941	0.014	280	[76]
**14c**	MeOH	336, 373, 392, 433, 511, 534s	720, 782, 882, 940	0.015	290	[76]
**15a**	H_2_O	252, 306, 344, 370, 441, 530, 570	728, 790, 900	0.084	2340	[77]
**15b**	H_2_O	253, 306, 345, 369, 441, 532, 570	727, 789, 900	0.082	2160	[77]
**16a**	MeOH	263, 298, 338, 367, 430, 475, 505, 525	717, 784, 885	0.017	410	[80]
**16b**	MeOH	263, 307, 344, 380, 430, 445, 525, 541	720, 783, 890	0.023	580	[80]

**Table 5:** Photophysical properties of **17**–**20b**.

Complexes	Solvent	λ_abs_/nm	λ_emi_/nm	Φ_PL_	Φ_Δ_	Lifetime/ns	Ref.
**17**	CH_2_Cl_2_	331,385,413, 437, 490, 540, 756	800, 915, 970	0.0017	0.56 (ACN)	360	[48]
**18**	CH_2_Cl_2_	275,352,537	650	0.35	0.73 (MeOH)	1940	[82]
**19**	ACN	/	650–750	0.058	/	/	[83]
**20a**	PBS	488	685	0.004	0.81	/	[84]
**20b**	PBS	488	685	0.005	0.76	/	[84]
**21**	PBS	370	686	0.197	/	1520	[85]

**Table 6:** Photophysical properties of **22a**–**29**.

Complexes	Solvent	λ_abs_/nm	λ_emi_/nm	Φ_PL_	Φ_Δ_	Lifetime/ns	Ref.
**22a**	Toluene	563, 606	624	0.399	0.53 (CH_2_Cl_2_)	106,600	[86]
**22b**	Toluene	610, 664	621, 691	0.132	0.81 (CH_2_Cl_2_)	156,500	[86]
**22c**	Toluene	596, 644	683	0.326	0.06 (CH_2_Cl_2_)	92,500	[86]
**22d**	Toluene	268, 729	684, 794	0.01	0.02 (CH_2_Cl_2_)	31,400	[86]
**23**	DMSO	352, 577, 629	642	/	0.06	4.70	[96]
**24a**	H_2_O	420; 518	657, 720	0.11	0.72	4.67	[87]
**24b**	H_2_O	427; 519	660, 724	0.05	0.89	4.82	[87]
**25a**	H_2_O	257, 417, 520, 558, 594, 650	656, 720	0.081	/	5.72	[97]
**25b**	H_2_O	256, 424, 521, 560, 594, 650	656, 720	0.036	/	5.87	[97]
**26a**	EtOH	293, 356, 510	652, 704	0.030	0.72 (PBS)	603	[88]
**26b**	EtOH	305, 356, 508	651, 705	0.018	0.29 (PBS)	408	[88]
**27a**	PBS	305	656	>0.01	<0.01	55 (93%) 281 (7%)	[89]
**27b**	PBS	550	615	0.004	<0.01	0.37 (73%) 3.3 (27%)	[89]
**28a**	H_2_O	685	/	<0.01	0.31	9.78	[32]
**28b**	H_2_O	678	/	<0.01	0.53	/	[32]
**29**	MeOH/H_2_O	814	1050	0.0017	0.146	/	[108]

## Data Availability

Not applicable.
